# Community response of soil microorganisms to combined contamination of polycyclic aromatic hydrocarbons and potentially toxic elements in a typical coking plant

**DOI:** 10.3389/fmicb.2023.1143742

**Published:** 2023-03-06

**Authors:** Qihui Shen, Wei Fu, Baodong Chen, Xuemeng Zhang, Shuping Xing, Chuning Ji, Xin Zhang

**Affiliations:** ^1^State Key Laboratory of Urban and Regional Ecology, Research Center for Eco-Environmental Sciences, Chinese Academy of Sciences, Beijing, China; ^2^University of Chinese Academy of Sciences, Beijing, China; ^3^RDFZ Chaoyang School, Beijing, China

**Keywords:** PAHs-PTEs, fungi, bacteria, community composition, molecular ecological network

## Abstract

Both polycyclic aromatic hydrocarbons (PAHs) and potentially toxic elements (PTEs) of coking industries impose negative effects on the stability of soil ecosystem. Soil microbes are regarded as an essential moderator of biochemical processes and soil remediation, while their responses to PAHs-PTEs combined contamination are largely unknown. In the present study, soil microbial diversity and community composition in the typical coking plant under the chronic co-exposure of PAHs and PTEs were investigated and microbial interaction networks were built to reveal microbial co-occurrence patterns. The results indicated that the concentrations of PAHs in the soil inside the coking plant were significantly higher than those outside the plant. The mean concentration of ∑16PAHs was 2894.4 ng·g^−1^, which is 5.58 times higher than that outside the plant. The average Hg concentration inside the coking plant was 22 times higher than the background value of Hebei province. The soil fungal community inside the coking plant showed lower richness compared with that of outside community, and there are significant difference in the bacterial and fungal community composition between inside and outside of coking plant (*p* < 0.01). Predicted contribution of different environmental factors to each dominant species based on random forest identified 20 and 25 biomarkers in bacteria and fungi, respectively, that were highly sensitive to coking plant soil in operation, such as *Betaproteobacteria，Sordariomycetes and Dothideomycetes*. Bacterial and fungal communities were shaped by the soil chemical properties (pH), PTEs (Hg), and PAHs together in the coking plant soils. Furthermore, the bacterial and fungal interaction patterns were investigated separately or jointly by intradomain and interdomain networks. Competition is the main strategy based on the co-exclusion pattern in fungal community, and the competitive relationship inside the coking plant is more complex than that outside the plant. In contrast, cooperation is the dominant strategy in bacterial networks based on the co-occurrence pattern. The present study provided insights into microbial response strategies and the interactions between bacteria and fungi under long-term combined contamination.

## 1. Introduction

The coking process in coke plants produces a variety of pollutants such as phenol, ammonium sulfate, polycyclic aromatic hydrocarbons (PAHs) and potentially toxic elements (PTEs), leading to soil contamination inside and outside the plant sites (Song et al., 2021). Of these, PAHs are persistent soil pollutants with significant toxicity, carcinogenicity, and mutagenicity (Wilcke, 2007; Han et al., 2017), and the 16 typical PAHs have been listed as priority pollutants by the United States Environmental Protection Agency (USEPA; Keith and Telliard, 1979). Long-term exposure to PAHs markedly increases the incidence rates of asthma, bronchitis, and heart disease (Kramers and Vanderheijden, 1988; Cai et al., 2017) and the risks of bladder, gastrointestinal, lung, and skin cancers (Ferreira-Baptista and De Miguel, 2005; Feng et al., 2009; Shen et al., 2014; Abdel-Shafy and Mansour, 2016). In addition, PTEs are gradually released and widely present in the soil environment as raw materials, intermediate or end products of industrial processes such as smelting, and fossil fuel combustion, which not only damage soil quality, but also increase the risk of bio-accumulation and skin absorption through human consumption and intake (Huang et al., 2019; Zeng et al., 2019), leading to a large number of serious health problems in many areas (Zhao H R, et al., 2012; Hu and Cheng, 2016). Trace concentrations of PTEs and PAHs in the soil can pose a threat to microorganisms, and bring soil ecological risks (Kashyap et al., 2019; Zhang et al., 2021; Zhao et al., 2021). Therefore, the management and rehabilitation of soil PAHs and PTEs in these sites have received enormous attention from many researchers and government (Balachandran et al., 2012; Li et al., 2020).

As a pollution absorbent, the soil has the capacity to hold a variety of pollutants, including pesticides, PTEs, and PAHs (Wolejko et al., 2020). Soil microorganisms can be regarded as important ecological indicators to evaluate soil contamination owing that they are sensitive to the pollutants and the changes in soil environment (Deng et al., 2015; Wang M, et al., 2019). Over the past decades, most of the studies on the response of microbial community to soil contamination focused on the effects of a single pollutant. Studies have reported soil microbial community abundance, population diversity and metabolic activity can be changed in response to PTEs (Beattie et al., 2018; Lin et al., 2019; Zhao et al., 2020). Furthermore, soil contamination with PAHs has been reported to decrease soil microbial diversity, abundance and metabolic functions (Gupta et al., 2019). However, environmental contaminants do not exist in isolation (Feron and Groten, 2002). Combined pollution is the most prominent feature of smelting polluted soil (Xu et al., 2020). It still remains unclear of how microbial diversity, community composition and co-occurrence patterns respond to long-term soil complex contamination. Therefore, the investigation of microbial community under long-term combined contamination under field condition is of significance for evaluating soil health and developing effective *in situ* bioremediation technologies.

So far, most investigations on the impact of compound pollutants on microorganisms have primarily concentrated on bacterial communities (Jiang et al., 2017; Liu et al., 2019). Zhao et al. found different responses of soil bacteria to PAHs-PTEs pollution at different depths in a steel mill with significant vertical changes in bacterial community structure (Zhao et al., 2021). It is generally accepted that microorganisms do not live in isolation, but form various ecological networks (Wolejko et al., 2020). They maintain the efficient utilization of resources and the balance of micro-ecosystem as a whole (Thavamani et al., 2012; Banerjee et al., 2019). Recently, studies have shown that fungi also play important roles in the interdomain molecular ecological networks of soil ecosystem (Geng et al., 2022). However, fungal community response to combined contamination still remains poorly understood. Furthermore, the response of both bacteria and fungi to industrial contamination has rarely been studied. The shifts in fungal and bacterial community in coking contaminated soils might reflect the environmental toxicity of PAHs-PTEs complex contamination and support soil microbe to be a potential indicator of the soil quality.

In recent years, intradomain and interdomain molecular ecological network analysis, as a sensitive and powerful tool, has demonstrated prominent advantages in revealing the interaction between microorganisms and environmental stresses (Deng Y, et al., 2012; Feng et al., 2019), Species in the same module had strong interaction or shared niche and microorganisms in a specific niche form complex interaction, which can be demonstrated by the co-occurrence network with species as nodes and associations as edges. The positive links indicate cooperation, while negative correlation might display competition and confrontation (Barberan et al., 2012; Deutschmann et al., 2021). Furthermore, network topological features can statistically determine the keystone taxa, which contributed to ecosystem stability (Berry and Widder, 2014; Shi et al., 2016; Banerjee et al., 2019). Network analysis have been applied to explore complicated microbial interactions for different ecosystems such as the rhizosphere (Fu et al., 2022), agricultural soils (Zhang X M, et al., 2022) and even human bodies (Duran-Pinedo et al., 2011), but few studies have researched the interactions of fungi and bacteria independently or jointly concerning coking impacted soils. The construction and analysis of the network of dominant species might help to decipher the structural changes and response mechanisms of microbial communities under long-term combined contaminations (Chen et al., 2020; Wu et al., 2021; Zhao et al., 2021).

In this study, we collected soil samples from inside and outside of a typical coking plant that has operated for 15 years. Soil bacterial and fungal communities were analyzed by high-throughput sequencing and modelling. The objectives of the study were to (i) compare the differences in soil microbial diversity and community composition and structure inside and outside of the coking plant; (ii) excavate biomarkers in soil microbes under long-term PAHs-PTEs contamination; (iii) elucidate the intradomain and interdomain interactions of soil bacterial and fungal communities in response to combined contamination. The study would allow a better understanding of the co-occurrence patterns of both bacterial and fungal communities under complex contamination, and may provide insights into microbial response strategies and the interactions between bacteria and fungi under long-term combined contamination.

## 2. Materials and methods

### 2.1. Site description and sample collection

Tangshan is the largest gathering city of coking plants in Hebei Province of China which produced a variety and large number of PAHs and PTEs every year. The surface soil samples (0–10 cm) were collected from inside (IN) and outside (OUT) of a typical coking plant in Tangshan (39°40′13.07″N, 118°21′56.25″E), which has been in operation for about 15 years by the time of sampling (Supplementary Figure S1A).

Samples inside the coking plant were collected by soil auger along the coking production line, including the coke-making and coke-quenching area, gas production area, and crude benzene production area. Meanwhile, soil samples outside the coking plant were collected from agricultural land, near the tree planting area and roadside. Together, a total of 35 soil samples [13 samples from inside (IN) and 22 from outside (OUT) of the coking plant] were collected (Supplementary Figure S1B). Five soil cores from each sampling point were mixed into one composite sample and sealed in brown ground-glass stoppered bottles. They were immediately transported to the laboratory on ice and sieved through a 2-mm mesh.

### 2.2. Soil physico-chemical analysis

Soil pH was measured by a PB-10 pH meter (Sartorius, Gottingen, Germany), and the soil/water ratio was 1:2.5 (m/v). Soil EC value was determined by using CON30EC meter (Shanghai Zhenmai Instrument Equipment Co., Ltd.), and the soil/water ratio was 1:5 (m/v). Before the determination of soil total carbon (TC), total nitrogen (TN) and total sulfur (TS),the soil samples were milled and parceled by tinfoil, and then the samples were put into the elemental analyzer (Vario El III, Elementar, Hanau, Germany) to determine the TC, TN and TS contents. Soil organic matter (OM) was measured by the ignition method. Soil available P (AP) was extracted by using 0.5 mol l^−1^ sodium bicarbonate (NaHCO_3_, pH 8.5) and measured with a microplate reader at 880 nm (PerkinElmer; Olsen, 1954). Soil moisture content measured by oven-dry method.

### 2.3. PAHs and PTEs determination

The concentration of PAHs was measured by gas chromatography–mass spectrometry method from the environmental protection standards of People’s Republic of China (HJ 805–2016; Ministry of Environmental Protection, 2016). Two grams of diatomaceous earth were completely mingled with 10 g soil sample in an accelerated solvent extraction (ASE 350) vessel. Then the samples were extracted by the Dionex ASE 350 accelerated solvent extraction system (Thermo Fisher Scientific, Waltham, MA, United States) using the extraction solution (acetone: n-hexane = 1: 1 v/v). The extracts were concentrated to 2 ml by using the parallel evaporator (Interface I-300, BUCHI, Germany). After purification with a magnesium silicate pure cation cartridge, the analytes were determined by gas chromatography–mass spectrometry detector (Agileng 6,890 N GC-5975C MSD Agilent Technologies, United States). Twenty percent of the soil samples were used for simultaneous analysis of duplicate samples, and the relative standard deviations (RSDs) of these duplicates were less than 10%.

The concentrations of potentially toxic elements (As, Cd, Cr, Cu, Ni, Pb, Zn, and Hg) of soil samples were determined by the following methods. Briefly, 0.5 g of air-dried soil samples were put into a polytetrafluoroethylene microwave digestion tube through a 100-mesh sieve. Three microliters of hydrofluoric acid, 5 ml of concentrated nitric acid, and 1 ml of hydrogen peroxide were added and shaken gently until they were well mixed. After inserting the digestive tube tightly, put it into the microwave digestion apparatus for digestion. Finally, the contents of As, Cd, Cr, Cu, Ni, Pb, and Zn in soil were ascertained by inductively coupled plasma mass spectrometry (ICP-MS Perkinelmer, Waltham, MA, United States). Hg was detected by an emergency portable mercury meter (RA-915 M, LUMWX, Canada). Blanks and internal standards were used to assure the accuracy of chemical analysis (GBW07429, China Standard Research Center).

### 2.4. DNA extraction, PCR amplification and sequencing

The soil genomic DNA was extracted by the PowerSoil DNA Isolation Kit (Qiagen, Germany) following the manufacturer’s instructions. The extracted DNA was amplified by the 16S rRNA universal primer set, 515 forward (5’-GTGCCAGCMGCCGCGGTAA-3′) and 806 reverse (5-'GGACTACHVGGGTWTCTAAT-3′) that targeted the V4 hypervariable regions of the prokaryotic 16S rRNA genes (Yarza et al., 2014; Ju et al., 2021). The ITS2 region of fungal ribosomal encoding genes was amplified with the primers gITS7 (5’-GTGARTCATCGARTCTTTG-3′) and ITS4 (5’-TCCTCCGCTTATTGATATGC-3′). The PCR reaction was executed with a total volume of 60 ul, which contained 6 μl 10x Ex Taq Buffer, 0.6 μl BSA, 6 μl dNTP, 0.3 μl Ex Taq, 1.2 μl of each primer, 1 μl DNA template, and 43.7 μl of H_2_O. Heated cycling conditions were as: 5 min at 94°C; 25 cycles of the 30s at 94°C, 30s at 55°C, 30s at 72°C; and a final step of 72°C for 7 min. Three technical repeats were performed for each test. The PCR products were integrated at equimolar concentrations, then sequenced on the HiSeq platform at MAGIGENE, Guangdong, China.

### 2.5. Bioinformatics and statistical analysis

The raw sequencing data were demultiplexed using an established sequence analysis pipeline integrated with various bioinformatics tools (Feng et al., 2017). Briefly, the reads were assigned to different samples according to the barcodes, allowing for a single mismatch. Next, primer sequences were trimmed and reads were joined by using FLASH (Magoc and Salzberg, 2011). The zero-radius OTUs (zOTU) as a form of amplicon sequence variants (ASVs) were generated using UNOISE 3 algorithm, and the chimera and low-abundance sequences (*n* < 8) were discarded (Magoc and Salzberg, 2011). To eliminate the influence of the difference in sequencing depth on downstream analyses, 58,631 and 40,000 reads were randomly resampled for bacteria and fungi of each sample, respectively. The rarefaction curves were further constructed for these normalized data. The resampled ASV tables were used for subsequent community analysis.

Principal Co-ordinates Analysis (PCoA) based on Bray-Curtis distance was carried out to visualize the compositional shifts of soil bacterial and fungal community, and the significance was tested using Permutational multivariate analysis of variance (PERMANOVA) in the vegan R package (Oksanen et al., 2007). Random forest algorithm was carried out using the randomForest R package (Liaw and Wiener, 2002). Variance partitioning was analyzed using the varpart function in the vegan R package (Oksanen et al., 2007). Data visualization was realized using the ggplot2 R package (Wickham, 2016).

### 2.6. Network construction and random forest modelling

The connections between interacting species can be used to explore the co-occurrence patterns of microorganisms and ecosystem stability. We constructed intradomain molecular ecological networks *via* a Random Matrix Theory (RMT)-based approach due to its advantage to automatically identify the appropriate similarity threshold prior to network construction (Deng Y, et al., 2012). ASVs were filtered by occurrence and only those existing in more than 80% of the total samples were kept for intradomain network computation. Asymmetric correlation matrix is calculated based on Spearman correlation coefficient and then converted into the similarity matrix by taking the absolute values. The data were filtered according to the relative abundance of OTU >0.05%, and the correlation threshold for network analysis was set at *r* > 0.6, *p* < 0.05. Spearman’s analysis method was used to construct the environmental factor-microbe co-occurrence network. Interactions with correlation coefficients no greater than 0.86 and 0.79 and *p*-values no less than 0.05 were excluded for bacterial and fungal communities, respectively. In addition, to check the influence of network samples on the topological nature of the network, 13 samples were randomly selected from 22 soil samples outside the coke plant to construct a microbial co-occurrence network with soil samples inside the plant. The data were filtered according to the relative abundance of OTU > 0.05%, and the correlation threshold for network analysis was set at *r* > 0.6, *p* < 0.05. Meanwhile，spearman’s analysis method was used to construct the environmental factor-microbial co-occurrence network.

To elucidate associations between fungi and bacteria in soil, interdomain network analysis *via* SparCC was carried out on the Galaxy-IDENAP platform (Friedman and Alm, 2012; Feng et al., 2019).^1^ Only those ASVs existing in more than 80% of the total samples were kept. Then we removed data with the absolute value of correlation coefficient smaller than 0.5 and filter the non-correlated associations with 0.05 significance. The obtained adjacent matrix associated with the bipartite graph consisted of 1 or 0, showing presence/absence of corresponding fungi-bacteria association. The topological properties like node degree, links, and modularity were calculated to explore alterations in associations between fungi and bacteria in the soil under permanent PAHs-PTEs stress. The keystone species were identified by the Zi-Pi plot based on the nodes’ roles within their network. All constructed networks were visualized using the interactive platform Gephi 0.9.2.

To obtain the best discriminant performance for taxa between inside and outside of coking plant, we regressed the relative abundance of bacteria and fungi using the RF algorithm to establish a model to correlate soil microbial community composition. Random Forest (RF) is one of the most robust ensemble machine learning classifiers and is unexcelled in accuracy among current algorithms for classification and regression (Liaw and Wiener, 2002; Friedman and Alm, 2012). The RF analysis (rfPermute function in rfPermute package in R) and the multiple regression model (lm function in stats package in R) were used to estimate the importance of different influencing pollutants (Jiao et al., 2022). Furthermore, we constructed an RF model to identify the most distinguished taxa from inside and outside of coking plant samples and test the accuracy of the biomarkers. Significantly enriched or depleted ASVs of bacteria and fungi were designated as biomarkers and the RF package v.4.6–14 was used with default parameters (Zhang et al., 2019; Ju et al., 2021).

### 2.7. Data availability statement

The datasets presented in this study can be found in online repository Genome Sequence Archive (GSA) with accession number: SUBCRA010697 and SUBCRA010706.

## 3. Results

### 3.1. Soil PAHs and PTEs concentrations

The concentrations of PAHs in the soil samples inside the coking plant were significantly higher than those outside the plant, and the concentration of PTEs inside and outside of the plant were not significantly different (Supplementary Figures S3, S4). Concentrations of almost all pollutants inside the coking plant, especially PAHs, exceeded the soil environmental quality of China-Risk control standards (GB 36600–2018): Class I land standards. The∑16PAH concentrations of soil samples inside coking plant were 70–17,000 ng·g-1, and the mean concentration was 2894.4 ng·g-1, which is 5.58 times higher than that outside the plant. The highest concentrations of all 16 PAHs inside the coking plant were Dibenzo (a, h) anthracene (DBA) and Chrysene (Chr). Compared with GB 15618–2018 (Soil environmental quality Risk control standard for soil contamination of agricultural land), the soil outside the coke plant is also slightly contaminated with PAHs. The concentrations of Hg in the soil inside and outside the coking plant far exceeded the background soil content values of Hebei province (Supplementary Table S1). In particular, the average Hg concentration inside the coking plant was 22 times higher than the background value. The physical and chemical parameters of 35 soil samples were reported in Supplementary Table S2.

### 3.2. Diversity and community composition of soil microorganisms

**Figure 1:**
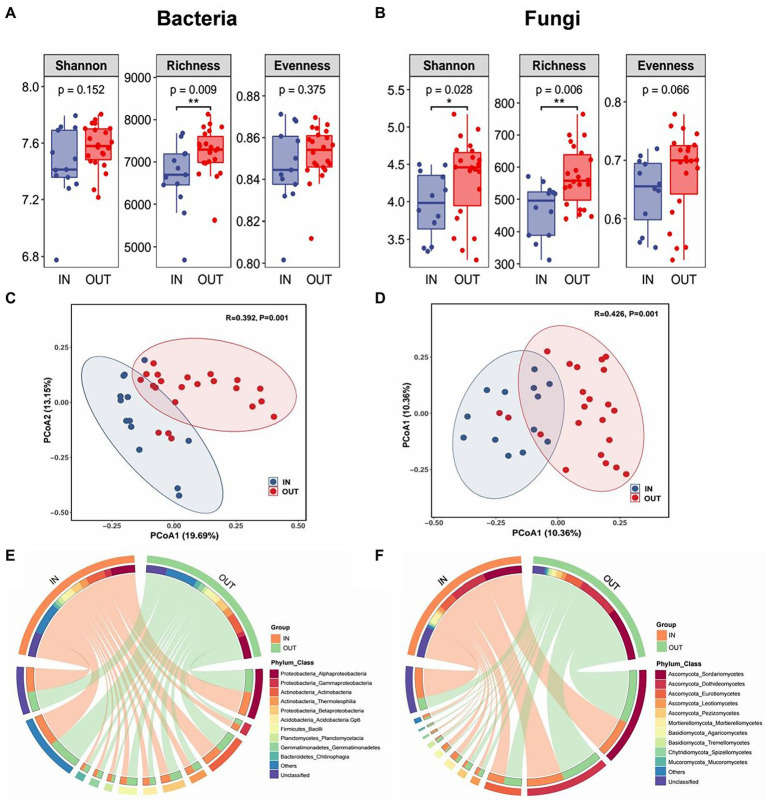
Box plots showed three alpha diversity indexes and their significant difference test results of soil bacterial community **(A)** and fungal community **(B)** inside (IN, blue) and outside (OUT, red) the coking plant. * and ** indicate significant differences between two sample areas based on the student-t test at confidence levels of *p* < 0.05 and *p* < 0.01, respectively. Unconstrained principal coordinates analysis (PCoA) showing that the inside and outside soil of the coking plant have significantly distinct bacterial **(C)** and fungal **(D)** community as detected by permutational multivariate analysis of variance (PERMANOVA). Circos plots shows the relative abundance of top 10 species at the class level (inner circle) and their corresponding phyla (outer circle) of bacteria **(E)** and fungi **(F)** in the two sampling areas.

Shannon-Wiener, Shannon’ s richness and evenness indexes were used to evaluate the α-diversity of soil microbial communities. The results showed that Shannon and Richness indexes of the fungal community inside the coke plant were significantly lower than that outside of the plant, but evenness of fungi was not significant. While for the bacterial community, only richness indicators were significantly lower inside the plant than that outside of the coking plant (Figures 1A,B). Based on PCoA and PERMANOVA, clear separations were observed along the first two PCoA axes in bacteria and fungi that explained 32.84 and 20.72% of total variance respectively, and the dissimilarities of corresponding microbial community were all significant (Figures 1C,D). In addition, the top 10 taxa at the class level were defined as the dominant or abundant species inside and outside of the coking plant, which accounted for 63–67% and 77–90% of the total abundance of bacteria and fungi, respectively (Figures 1E,F). Specifically, *Alphaproteobacteria* and *Actinobacteria* are the top two classes of bacteria in the relative abundance, accounting for about 16 and 13% of the dominant species respectively, and there are only minor differences between the dominant species between inside and outside of coking plant. As for fungal community, the relative abundance of *Mortierellomycetes*, *Agaricomycetes,* and *Spitellomycetes* were substantially higher inside the coking plant compared with that outside the plant.

**Figure 2:**
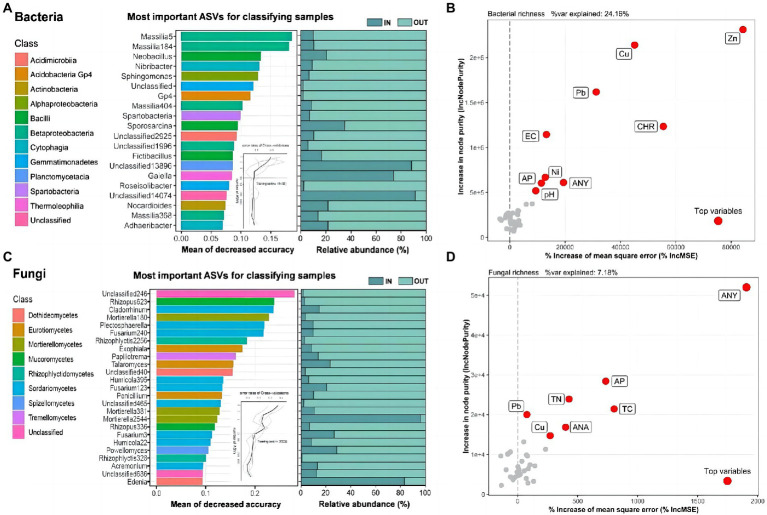
Microbial biomarkers between soil samples inside and outside the coking plant. The top 20 and 25 ASVs of bacteria **(A)** and fungi **(C)** are identified by Random-forest model in the two sampling areas and annotated at the genus and class level. These ASVs are ranked in descending order of importance to the accuracy of the model. The curve inset represents 10-fold cross-validation error as a function of the number of input ASVs used to differentiate inside and outside soil microbiota ranked by variable importance. The corresponding bar plots show the relative abundances of the biomarkers. Random forest analysis showing the relative contribution of biotic and abiotic factors in predicting bacterial **(B)** ASVs and fungal **(D)** ASVs richness response to coking plant (the more important the index, the greater the values). Abiotic variables: polycyclic aromatic hydrocarbons (PAHs), potential toxic elements and soil properties.

The 20 and 25 most significant ASVs were designated as biomarkers (Figures 2A,C), some of which contained unclassified taxa, and the RF model created by the RF algorithm explained 92.50 and 85.60% of the bacterial and fungal variance related to inside and outside of the coking plant separately at the class level. The genus *Massilia* in class *Betaproteobacteria* was the most important and common biomarker, accounting for 1/5 of the total bacterial biomarkers (Figure 2A). As to fungi, about 1/3 of the biomarkers belonged to class *Sordariomycetes* (Figure 2C), while the most interpreted biomarker was unclassified. The results showed that most important biomarkers of both bacteria and fungi outside of the coking plant had a much higher the relative abundance than that inside of the plant (Figures 2A,C). On the contrary, except for two unclassified genera, only the *Gaiella* in the bacterial community and *Mortierella* and *Edenia* in the fungal community owned higher relative abundance in the coking plant compared with those outside the plant under combined contamination. We further explored variables affecting soil bacterial and fungal diversity using the random forest algorithm (Figures 2B,D). For soil microbial richness, the results showed that potentially toxic elements and PAHs are the top explaining variables.

### 3.3. Relationship between the environmental variables and dominant species

**Figure 3:**
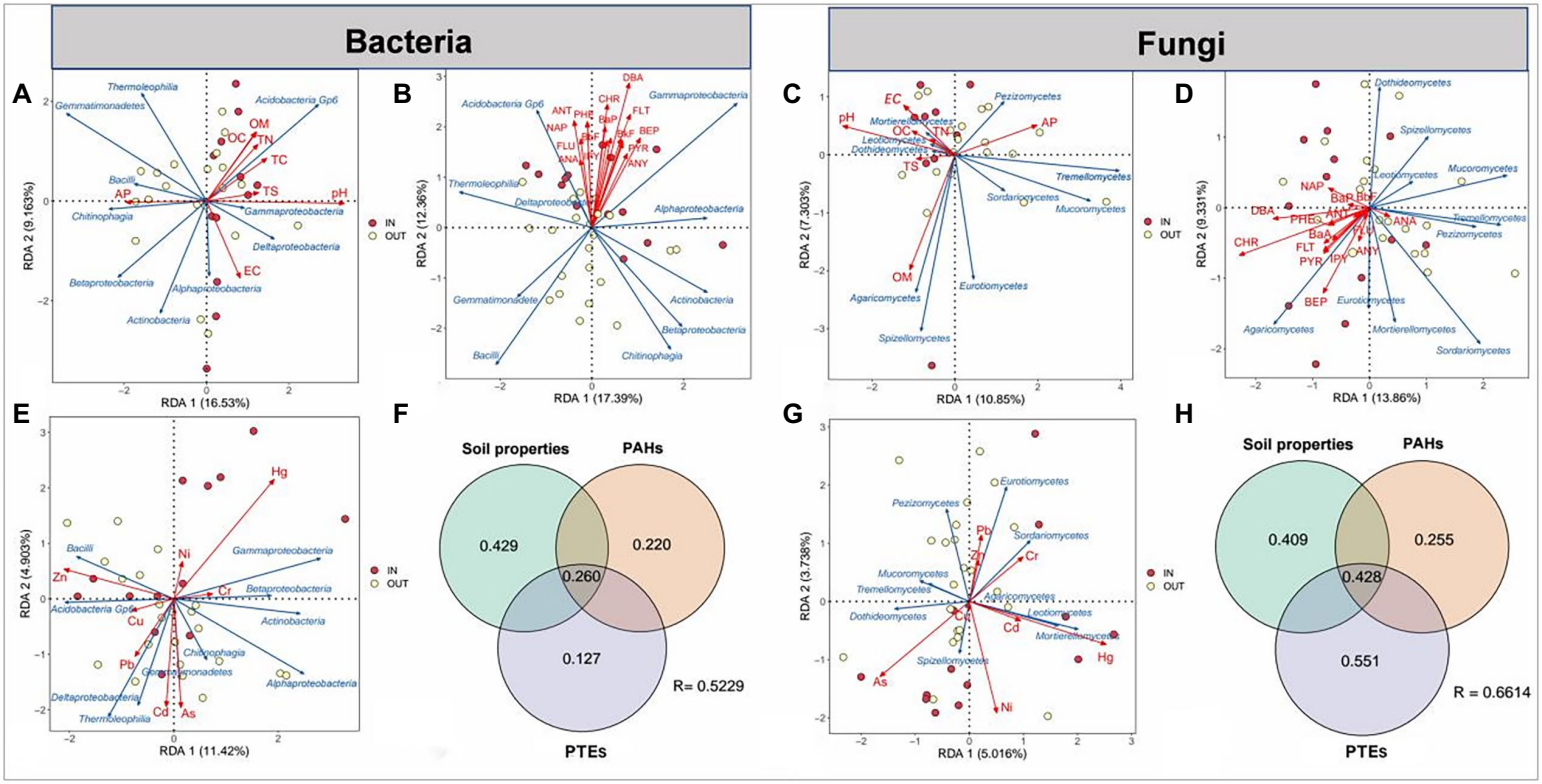
Redundance analyses (RDA) of top 10 species of the bacterial and fungal communities at the class level (blue arrows) and three groups of environmental factors (red arrows) in the soil samples inside (IN, red dots) and outside (OUT, yellow dots) the coking plant. The environmental factors include eight soil physicochemical properties **(A,D)**, 16 polycyclic aromatic hydrocarbons (PAHs) **(B,D)** and eight heavy metals **(E,G)**. The contributions (%) of three groups of environmental factors are identified *via* variance decomposition analysis (VPA) in the variation of bacterial **(D)** and fungal **(H)** community structure.

Redundance analyses (RDA) and variance decomposition analysis (VPA) were used to further explore the associations between three environmental factors (PAHs, PTEs, soil properties) and the community structure of top 10 microbial species at the class level (Figure 3). RDA analysis showed that pH was the most influential environmental parameter for bacteria, while OM was the most important environmental factor for fungi in 8 soil properties. The obvious cluster of 16 PAHs showed that they had similar distribution on microbes, among which DBA and Chr had the greatest relevance with bacterial and fungal communities, respectively (Figures 3B,D). For PTEs, zinc (Zn) and Hg had the greatest influence on bacteria community composition, while Hg and arsenic (As) were the most predominant factor for fungal community. These results indicated that different dominant microbial species have obvious preferences to different PTEs contamination. For example, Zn content was positively correlated with *Bacilli* and *Acidobacteria Gp6* the relative abundance and negatively with *Gammaproteobacteria* and *Betaproteobacteria* in bacterial the relative abundance, whereas soil Hg content showed opposite effects on the abundance of these bacteria (Figure 3E). Similarly, the same pattern was observed in fungi. The effect of Hg on the relative abundance of *Mortierellomycetes* and *Leotiomycetes* was positively correlated, while the effect of As on them was negatively correlated (Figure 3G).

Subsequent VPA showed that the environmental factors with the highest explanation ratio for fungi were PAHs-PTEs combined contamination, accounting for about 0.806 of the total variation. In addition, the effect of soil properties on the bacterial community was 0.429 (Figures 3F,H). These results indicated that PAHs, PTEs and soil properties played important roles in shaping the microbial communities.

### 3.4. Microbial interaction patterns and hubs of intradomain and interdomain networks

**Figure 4:**
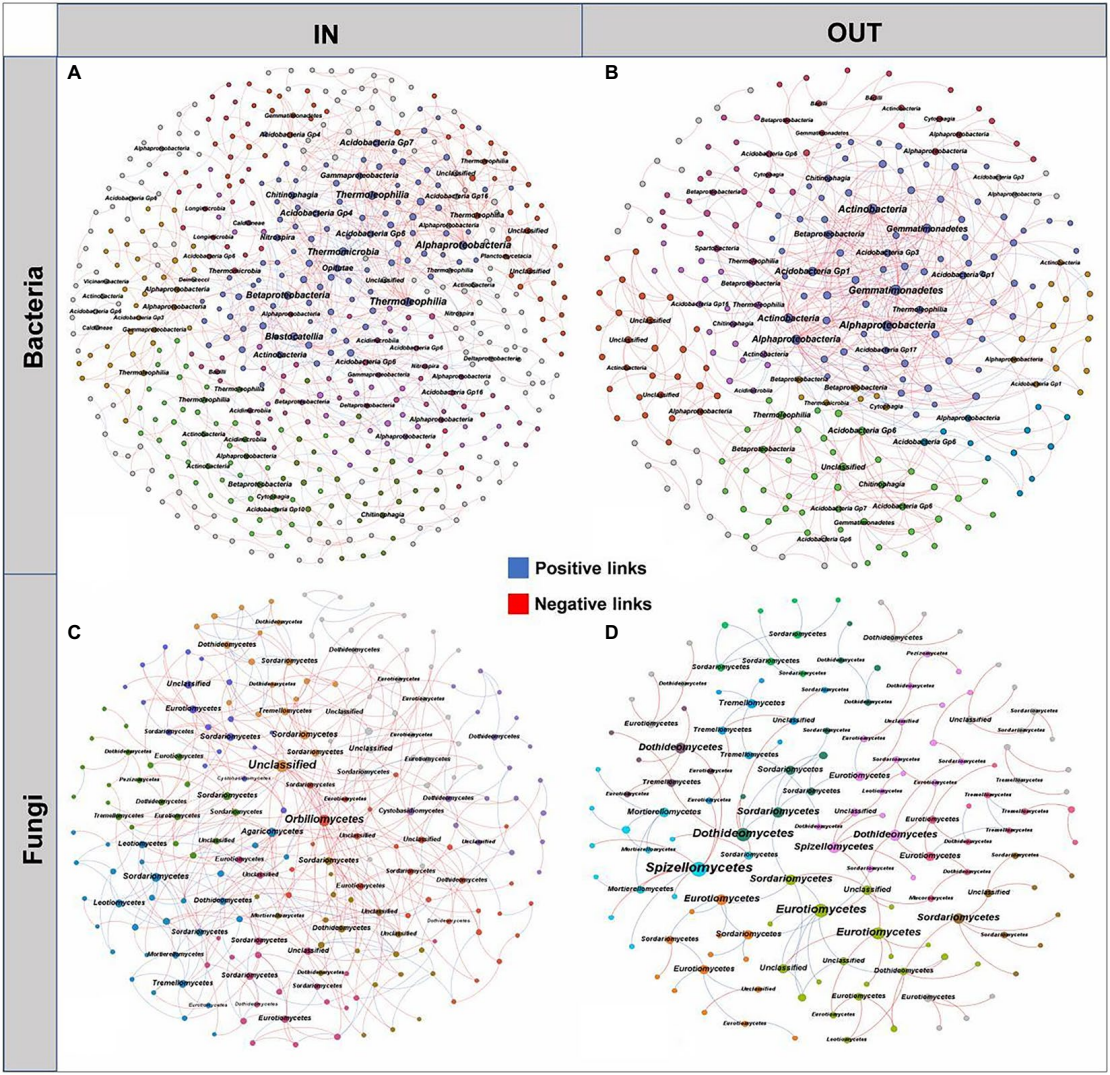
The networks of soil bacterial **(A,B)** and fungal **(C,D)** communities inside and outside the coking plant. The networks are colored by different main modules, which means the nodes clustered in the same module share the same color. The node size is weighted based on node degree and main nodes degree in each module are annotated at the class level. The blue and red links represent positive and negative correlations, respectively.

The microbial interaction patterns were explored *via* molecular ecological networks and their global topological properties inside and outside of the coking plant. In general, the modularities of both bacterial and fungal networks were relatively high (>0.60), while bacterial networks had more nodes and links than fungal networks (Figure 4). It was noteworthy that the relationships in fungal community tended to be co-exclusion rather than co-occurrence, with more than half of fungi being negatively correlated, while the opposite pattern was observed in bacterial networks. As for the fungal network, there were more nodes and connections inside the coking plant than that outside, indicating that the fungal network inside the plant was more complex than that outside the plant (Figures 4C,D). Among them, the network relationships in the fungal community were mainly negative, and there were more negative links inside the plant than the outside (68.28% > 59.62%; Supplementary Table S2). Similarly, there are more nodes and connections for the bacterial microbial networks inside the coking plant than the outside, indicating that the bacterial network inside the plant was more complex than that outside the plant (Figures 4A,B). Differently from fungal network, the network relationships in the bacterial community were predominantly positive, and the positive links inside the plant were less than those outside the plant (72.50% < 82.84%; Supplementary Table S3). Besides that, the network associations of bacterial and fungal communities inside the coking plant are more connected, characterized by smaller modularity and higher clustering coefficient (avgCC), average connectivity (avgK) and shorter harmonic geodesic distance (HD; Supplementary Tables S2, S3). In conclusion, the soil microbial community inside the coking plant showed a more complex and tighter network structure than the outside. This is similar to the results of constructing a microbial occurrence network with 13 randomly selected soil samples from outside the plant and inside the plant (Supplementary Figure S5; Table S4). We have constructed the network of bacterial and fungal co-occurrences inside the coking plant with PAHs-PTEs and soil variables (Supplementary Figure S7). Our results show that inside of plant soil microbes (both bacteria and fungi) were highly connected with PAHs-PTEs contamination.

Moreover, the microbial species with higher node degrees in each module were screened as hubs and annotated at the class level (Figure 4). The results showed that there were different bacterial and fungal hubs between inside and outside of coking plant. Specifically, for the bacterial networks, the hubs shared by inside and outside of the coking plant mainly included *Alphaproteobacteria* and *Acidobacteria*, while more hubs of *Betaproteobacteria* and *Thermoleophilia* were found in the bacterial network inside of plant (Figures 4A,B). For the fungal networks, the hubs shared by inside and outside of the coking plant mainly included *Eurotiomycetes*, *Dothideomycetes,* and *Sordariomycetes*, and the network inside the plant had more *Orbiliomycetes*, *Agaricomycetes*, *Tremellomycetes* and unclassified microbes as the hubs (Figures 4C,D).

**Figure 5:**
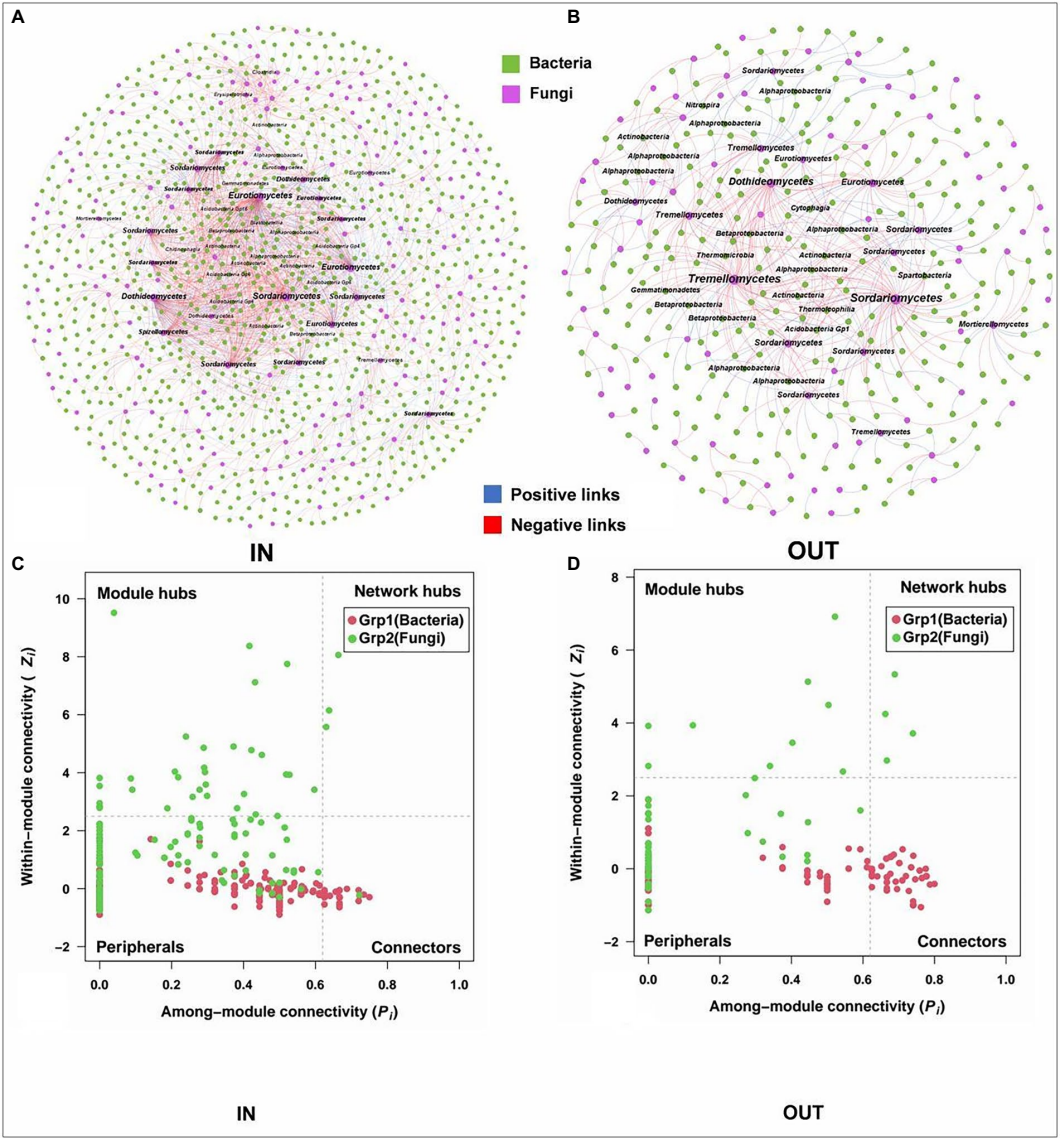
Interdomain ecological networks of the inside bacterial-fungal associations of inside **(A)** and outside **(B)** and their Zi-Pi plots of the soil samples inside **(C)** and outside **(D)**. A connection stands for a strong (Spearman’s ρ > 0.70) and significant (*p* < 0.05) correlation. The networks are colored by the domains. Node sizes are associated with node degree and about top 5% nodes of degree in each module are annotated at the class level. The blue and red links represent positive and negative correlations, respectively. The Zi-Pi plots exhibit the distributions of ASVs based on their topology. The threshold values of Zi and Pi for categorizing ASVs are 2.5 and 0.62, respectively.

The bacterial-fungal interaction patterns and hubs were revealed by interdomain networks (Figure 5). The network of the inside coking plant owned more nodes and links compared to those outside the coking plant with the same strong (Spearman’s ρ > 0.70) and significant (*p* < 0.05) correlation. Clearly, the negative associations dominated the bacterial and fungal interaction patterns, and the relationship was further strengthened in the network inside the coking plant. In addition, node sizes are weighted according to node degree, with the top 5% of nodes in each module being considered hubs and annotated at the category level (Figure 5). Interestingly, fungal hubs had the densest network connectivity and higher node degree than bacterial hubs in the networks both inside and outside the coking plant, suggesting that fungi dominate microbial interactions in interdomain networks (Figures 5A,B). Specifically, the fungal hubs under contamination were mainly *Eurotiomycetes*, *Sordariomycetes,* and *Dothideomycetes*, which are the same hubs shared by bacterial or fungal intradomain networks, while the hubs outside of coking plant showed a higher diversity at the class level. Similar results were also observed in bacterial hubs of the interdomain network (Figures 4, 5). The bacterial-fungal associations in the networks were classified into four categories based on their within-module connectivity (Zi) and among module connectivity (Pi) values: peripherals, connectors, module hubs and network hubs (Figures 5C,D). The module hubs and connectors were proposed to be keystone taxa because they played important roles in network topology (Deng et al., 2011). Notably, all the keystones in the interdomain network belonged to the fungi (Figures 5C,D). Moreover, there were fungal keystones inside the coking plant network than that outside the plant (Figure 5C). Similar results are shown in Supplementary Figure S6.

## 4. Discussion

### 4.1. PAHs-PTEs combined contamination altered microbial diversity and community composition

It was found that higher concentration of combined contaminants inside the coking plant significantly reduced the diversity of fungal communities, and the structure of bacterial and fungal communities inside and outside the coking plant were significantly different. It is the long-term coking production process that resulted in the accumulation of PAHs and PTEs in the coking plant and thus made a significant difference in soil contaminant concentrations between inside and outside the coking plant. Consistent with numerous previous studies, PAHs and PTEs contamination would decrease microbial diversity (Moffett et al., 2003; Li et al., 2015; Wang X, et al., 2019; Jara-Yanez et al., 2021). On the one hand, microorganisms resist environmental contaminants through complex interactions to maintain the community stability (Barberan et al., 2012; Zhao et al., 2021; Zhang X, et al., 2022). On the other hand, the succession of microbial interaction from non-adaptive to adaptive pattern drives the change of microbial composition and structure under combined contaminations (Chen et al., 2020). Therefore, it was inferred that the decrease in microbial community richness under PAHs-PTEs contamination inside the coking plant may be a strategy for fungi and bacteria to maintain the community stability and resist combined contamination. Many studies have described a decline of bacterial α-diversity and its association with environmental factors under soil pollution (Thavamani et al., 2012; Yang et al., 2022; Zhang X, et al., 2022). Our results further indicated that soil bacterial and fungal communities under chronic PAHs-PTEs stress were significantly reduced in richness rather than evenness, and fungi showed greater variation in diversity and community composition compared to bacteria (Figure 1). The diversity and structural variation of fungal communities is more significant than that of bacteria inside the coking plant. We speculated that combined contamination in the field has a greater impact on fungal community. Alternatively, the fungal communities are more responsive and sensitive to environmental changes compared to bacteria. On one hand, it has been shown that fungi can use PAHs as a carbon source for growth and reproduction, thus increasing their species richness (Souza et al., 2016). However, the lower positive impact from PAHs may turn into a negative impact if PAHs concentrations exceed a certain turning point (Tsui et al., 1998), which is confirmed by the negative correlation between PAHs and most fungi (genera) in the present study. On the other hand, it has been reported that the growth of fungal hyphae penetrated the soil layer could be more easily accessible to pollutants, and thus had better tolerance to pollutants and could adapt to more complex pollution environments than bacteria (Harms et al., 2011; Zhao et al., 2018).

### 4.2. Different coking-tolerant biomarkers identified inside and outside the coke plant

Random Forest (RF) was regarded as a dependable method to select biomarkers in microbiome (Bruckner et al., 2017). Previous researches have shown the application of RF in detecting microbial biomarkers in PAHs and PTEs contaminated soils, respectively (Cipullo et al., 2019; Xie et al., 2020). Several reviews have summarized the functional microorganisms with PAHs or PTEs degradation potential (Liu et al., 2017; Ali et al., 2022). The RF results showed that the variance of soil bacterial and fungal richness were mainly explained by PAHs and PTEs, suggesting they were mainly influenced by pollutants but not soil properties in this study (Figures 2B,D). Therefore, we could claim that most of the biomarkers could represent changes of pollution levels to some extent. In the present study, the *Massili* of the class *Betaproteobacteria* in bacteria community might be a potential biomarker in combined contaminated soils and was supposed to show obvious PAHs-PTEs resistance (Figure 2A). According to several research, the class Betaproteobacteria is resistant to soil contamination (Lors et al., 2012; Das et al., 2013). Due to the heredity of the species and the result of long-term adaptation to the circumstances, *Betaproteobacteria* was tolerant to soil contamination (Zhu et al., 2013). Furthermore, the class *Sordariomycetes* and *Dothideomycetes* of fungal community were also regarded as biomarkers in PAHs-PTEs compound contaminated soils (Figure 2B). Carrie Sim et al. also discovered metal-tolerant endophytic fungi from the phytoremediator plant phragmites and found that six isolates belonging to *Sordariomycetes* and eight isolates belonging to *Dothideomycetes* were highly tolerant to soil pollution (Sim et al., 2018). Although Verma et al. reported that some taxa of biomarkers have PAHs or PTEs degradation ability (Verma and Kuila, 2019), they may maintain low levels of growth and metabolism while utilizing the pollutants. This implied that some microbes benefited from pollutant metabolism and achieve the shift from non-adaptive to adaptive taxa. Furthermore, the microbes with high sensitivity to environmental disturbance or stress can be used as ecological indicators to monitor soil contamination and provide a more comprehensive understanding of soil health. Hence, we identified 20 bacterial and 25 fungal biomarkers that responded strongly to the pollutants at the class and genus levels. Differences in the relative abundance of most biomarkers inside and outside the coking plant suggested that biomarkers tended to be” sensitive” to PAHs-PTEs stress and biomarkers inside the coking plant exhibit response strategy by reducing their abundance (Figure 2).Therefore, we extrapolated that PAHs-PTEs contamination markedly influenced soil bacterial and fungal community structures, and might decrease the relative abundance of tolerant biomarkers inside the coking plant. Taken together, these results deepened our understanding of differentiated microbial degradation capacity under long-term combined pollution.

### 4.3. Environmental variables and soil contaminants together drive the microbial community composition

It was reported that soil microbial community structures are highly sensitive to soil environmental changes and stresses (Zhao et al., 2021; Yang et al., 2022). The combined analysis of VPA and RDA showed that PAHs and PTEs played a crucial role in shaping the composition and structure of bacterial and fungal communities, and the effects of PTEs were greater than those of PAHs (Figure 3). Our results suggested that Hg affected the richness and structural composition of soil microbial communities, consistent with previous studies (Frossard et al., 2017; Luo et al., 2019). Different forms of Hg in the environment can be transformed by biotic and abiotic interactions, especially microorganisms that drive methylation, demethylation, reduction and oxidation of Hg under certain conditions, thus affecting the mobility and biological effectiveness of Hg (Siciliano et al., 2002; Barkay et al., 2005; Lu et al., 2016). In turn, Hg can also be enriched in organisms, thus driving changes in microbial community structure (Siciliano et al., 2002; Lehnherr and Louis, 2009; Qureshi et al., 2010). Moreover, different dominant microbial species have obvious preferences for different PTEs, for example, *Gammaproteobacteria* and *Betaproteobacteria* showed a positive correlation with Hg but a negative correlation with Zn. Previous studies have reported the degradation or sensitivity of these microorganisms to specific PTEs, which is an important reason for the adaptive changes in microbial community structure in soils contaminated with multiple PTEs for a long time (Tipayno et al., 2018; Chen et al., 2020; Zhao et al., 2021). In addition, the effects of 16 typical PAHs on microbial community structure tended to be consistent. This may be due to the fact that PAHs had similar condensed aromatic rings, thus exerting a selective pressure in approximately the same direction on the microbes, although to a different degree. However, different heavy metals show differentiated or even opposite effects, which may be attributed to the large differences in the concentrations of heavy metals utilized and tolerated by different microbial metabolisms, and these microbial characteristics are difficult to change under long-term PTEs stress (Giller et al., 1998; Yang et al., 2022).

In addition, soil properties also took a part in shaping the bacterial and fungal community structure (Figures 3A,E). Many previous studies have reported the relationships between microbial communities and soil properties (Zhao X G, et al., 2012; Li et al., 2017; Nam et al., 2021). In the present study, pH plays an essential role in shaping both bacterial and fungal communities among 8 soil properties, which was in accordance with previous reports (de Mora et al., 2005; Zhao et al., 2013; Carlson et al., 2019). pH can alter the solubility of organic carbons (Andersson et al., 2000), change microbial metabolic activities (Fang and Liu, 2002), and increase the metal bioavailability (Cipullo et al., 2018). The availability and mobility of contaminants in soils can be altered by soil pH (Calugaru et al., 2016; Pakzad et al., 2016), which might indirectly influence the fungal community structure. Furthermore, the soil pH as the major geochemical variable also significantly affected the bacterial community, which is in accordance with previous reported results (Calugaru et al., 2016; Pakzad et al., 2016). The apparent direct impact of pH on bacterial community composition is most likely owing to the restricted pH ranges required for optimum bacterial development (Rousk et al., 2010).

### 4.4. Co-occurrence patterns of microorganisms in PAHs-PTEs contaminated soils

Microbial interactions are fundamental to ecosystem function and could result from both ecological associations between microorganisms and abiotic environmental selection, while the molecular ecological networks under long-term PTEs-PAHs stress are poorly characterized (Deutschmann et al., 2021; Wu et al., 2021; Zhao et al., 2021). Although network analysis may not always indicate true biological connections (Blanchet et al., 2020), it has contributed to the understanding of the complex interrelationships of microbes and how they respond to multiple contaminants (de Vries et al., 2018; Banerjee et al., 2019). By comparing intradomain and interdomain network of bacteria and fungi alone or jointly between inside and outside the coking plant, we described the shifts in microbial interaction patterns on a whole and the hubs dominating the patterns (Figures 4, 5). The network of bacterial and fungal co-occurrences with PAHs-PTEs and soil variables show that inside of coking plant soil microorganisms (including bacteria and fungi) are strongly affected by contamination with PAHs-PTEs (Supplementary Figure S7). Our study showed that networks inside the coking plant under chronic PAHs-PTEs stress became more complex and had an increased proportion of negative interactions than that outside, which is consistent with the results of most previous studies on the effects of single pollutants (Zhang X, et al., 2022). The increase in hubs and negative interactions in the fungal network inside the coking plant suggested that competition is the main strategy. It is widely accepted that network stability increases with network complexity, especially in relative modularity, and increased negative interactions often imply enhanced microbial network stability (Montoya et al., 2006; Yuan et al., 2021). Furthermore, fungal communities are more resistant to environmental changes and can improve their tolerance to soil pollution by collaborating with plant roots (Zhang X M, et al., 2022; Zhang X, et al., 2022), so they maintain community stability with a competitive relationship. While the predominance of cooperation in bacterial and bacterial-fungal networks further supports the inference that bacteria and fungi have different adaptive mechanisms in response to the chronic pollution stress. Researches shows that although cooperating networks of microbes can be efficient, they are often unstable (Coyte et al., 2015; Hernandez et al., 2021). Since bacterial communities are more responsive to environmental stresses (Li et al., 2017) and the community structure is more inclined to respond to environmental stresses with cooperative relationships (Yang et al., 2018; Qi et al., 2022), our study further demonstrates that bacterial communities respond in a cooperative-dominated manner in response to combined soil contaminations. Moreover, documenting the co-occurrence patterns across complex and diverse communities may help to ascertain the functional roles or environmental niches occupied by uncultured microorganisms (Chaffron et al., 2010). A proportion of unclassified taxa were observed in the species composition, biomarkers and network hubs at the class and genus level, which suggested that they may also play an important role in maintaining the stability of microbial community. Hence, the isolation and cultivation of unknown taxa are imperative for a comprehensive knowledge of the response of microbial communities and pollution control.

The composition and assembly of the community will be affected by the keystone species in the entire microbial community (Berry and Widder, 2014). Without keystone species, the entire community would collapse, known as “ecosystem engineers” due to the critical role of keystone species in the microbial community (Banerjee et al., 2018). In this study, it is worth noticing that some different bacterial and fungal specific keystone taxa (*Betaproteobacteria, Thermoleophilia, Orbiliomycetes, Agaricomycetes* and *Tremellomycetes*) were discovered inside and outside the coking plant. Some keystone taxa proven in this study in PAHs-PTEs contamination groups have been shown to develop PAHs and PTEs removal and detoxification functions (Tancsics et al., 2010; Uyar et al., 2014; Tan and Parales, 2019). For example, isolated *Agaricomycetes* has great potential as a biosorbent to adsorb PAHs from industrial brownfields (Lemmel et al., 2021) and can be utilized for PTEs recovery from electronic-waste (Qi et al., 2022). Similarly, some members of *Agaricomycetes* displayed active roles in mitigating the stress of combined heavy metal toxicity on surface-sterilized rape roots (Deng Z J, et al., 2012). The potential keystone species may contribute to resisting environmental perturbations such as PAHs-PTEs contamination when the microbial community composition changed under environmental stress. Furthermore, keystone species can produce antibiotics or directly participate in synergistic connections that alter the microbial community composition. As a result, the microbial community can be influenced by keystone species in a number of ways, and the specific ways would vary depending on the environment (Dee et al., 2019). However, there was still very little information available in this study on the function of several keystone taxa, which need further investigations. Overall, PAHs-PTEs pollution which highly influenced soil microbial communities would modify the co-occurrence pattern of bacteria and fungi inside the coking plant, and the shift in the co-occurrence pattern was most likely a strategy to adapt to PAHs-PTEs contamination.

## 5. Conclusion

In this study, we found that long-term PAHs-PTEs complex pollution caused by the coking process inside the plant decreased soil microbial diversity. The soil fungal and bacterial community compositions were significantly different between sampling sites inside and outside of coke plant. Some coking-tolerant groups, such as *Betaproteobacteria, Sordariomycetes and Dothideomycetes* were enriched in coking contaminated soils, which might be considered as coking-tolerant biomarkers. Soil chemical properties (pH), PTEs (Hg) and PAHs together significantly affected the soil bacterial and fungal community structure in the coking plant soils. Moreover, to better adapt to combined contamination stress, the relationships in fungal community tended to co-exclusion rather than co-occurrence, with more than half of them being negatively correlated, while the opposite pattern was observed in bacterial networks. Some different bacterial and fungal specific keystone taxa inside and outside the coking plant, such as *Betaproteobacteria*, *Thermoleophilia*, *Orbiliomycetes*, *Agaricomycetes*, and *Tremellomycetes* were proved to develop the function of biosorption of PTEs-PAHs contamination, which might play a role in remediating coking contaminated soils. However, the specific ecological functions of the biomarkers and keystone genera should be further explored. In summary, our findings show the response and interaction patterns of soil microbial communities to long-term combined pollution at three levels: dominating microbial species at the class level, bacterial and fungal communities, and their interactions across domains. It is also important to mention that a microbiome comprises bacteria, archaea, and other members, but only soil fungi and bacterial community were considered in this study. It is recommended that future study should focus on critical functional genes linked with complicated pollution mitigation and *in situ* screening of bacteria and fungi with degrading capabilities for a wide variety of contaminants.

## Data availability statement

The datasets presented in this study have been deposited in the Genome Sequence Archive (GSA) (https://ngdc.cncb.ac.cn/gsa/) with accession numbers CRA007106 and CRA007108.

## Author contributions

QS: investigation, methodology, software, validation, formal analysis, data curation, visualization, and writing original draft. BC: conceptualization, resources, review and editing, and supervision. XuZ: methodology. SX: investigation. CG: data curation. XiZ: conceptualization, writing—review and editing, supervision, project administration, and funding acquisition. All authors contributed to the article and approved the submitted version.

## Funding

This study was financially supported by the National Natural Science Foundation of China (41977042, 42177109 and 21677164) and National Key Research and Development Program of China (2018YFC1800302).

## Conflict of interest

The authors declare that the research was conducted in the absence of any commercial or financial relationships that could be construed as a potential conflict of interest.

## Publisher’s note

All claims expressed in this article are solely those of the authors and do not necessarily represent those of their affiliated organizations, or those of the publisher, the editors and the reviewers. Any product that may be evaluated in this article, or claim that may be made by its manufacturer, is not guaranteed or endorsed by the publisher.

## Supplementary material

The Supplementary material for this article can be found online at: https://www.frontiersin.org/articles/10.3389/fmicb.2023.1143742/full#supplementary-material

## Footnotes

^1^ http://mem.rcees.ac.cn:8080/
