# Supplementary material for: Community response of soil microorganisms to combined contamination of polycyclic aromatic hydrocarbons and potentially toxic elements in a typical coking plant
Source: Front Microbiol. 2023 Mar 6;14:1143742. doi: 10.3389/fmicb.2023.1143742 (PMC10025358; doi:10.3389/fmicb.2023.1143742)
Supplement: Supplementary file 1 [file Data_Sheet_1.pdf]

Supplementary Material

Community response of soil microorganisms to combined contamination of polycyclic aromatic hydrocarbons and potentially toxic elements in a typical coking plant

Qihui Shen, Wei Fu, Baodong Chen, Xuemeng Zhang , Shuping Xing, Chuning Ji, Xin Zhang *

*** Correspondence:** Xin Zhang: xinzhang@rcees.ac.cn

Figures

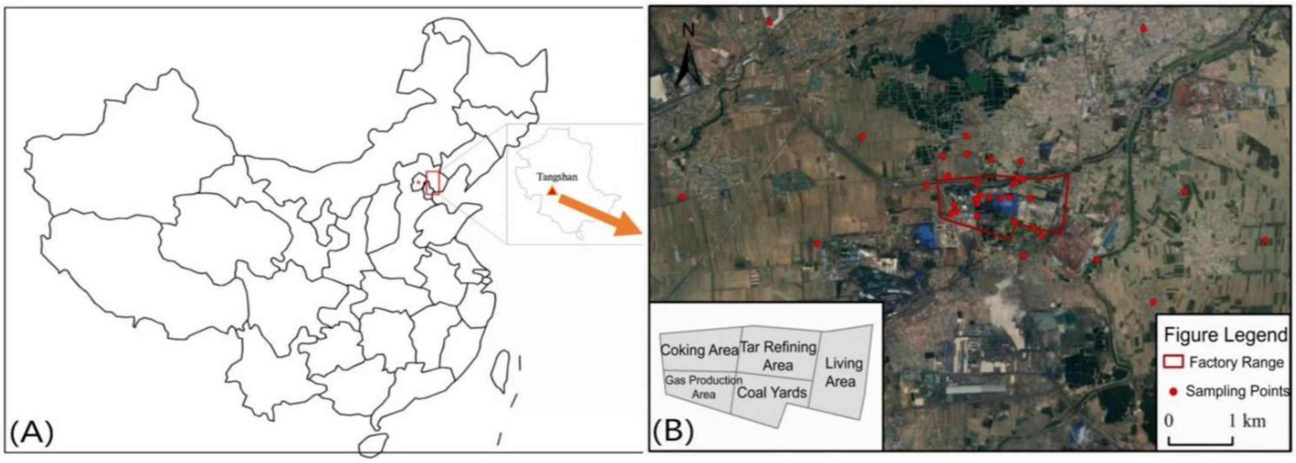

Fig. S1 Sampling sites inside and outside the coking plant in Tangshan city.

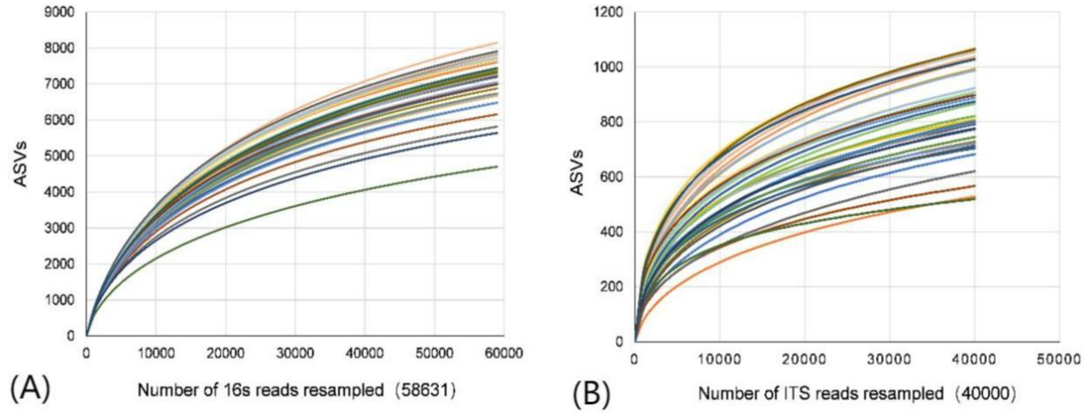

Fig. S2 Rarefaction curves represent the number of amplicon sequence variants (ASVs) depending on the number of 16S rRNA genes (a) and ITS (b) sequences amplified.

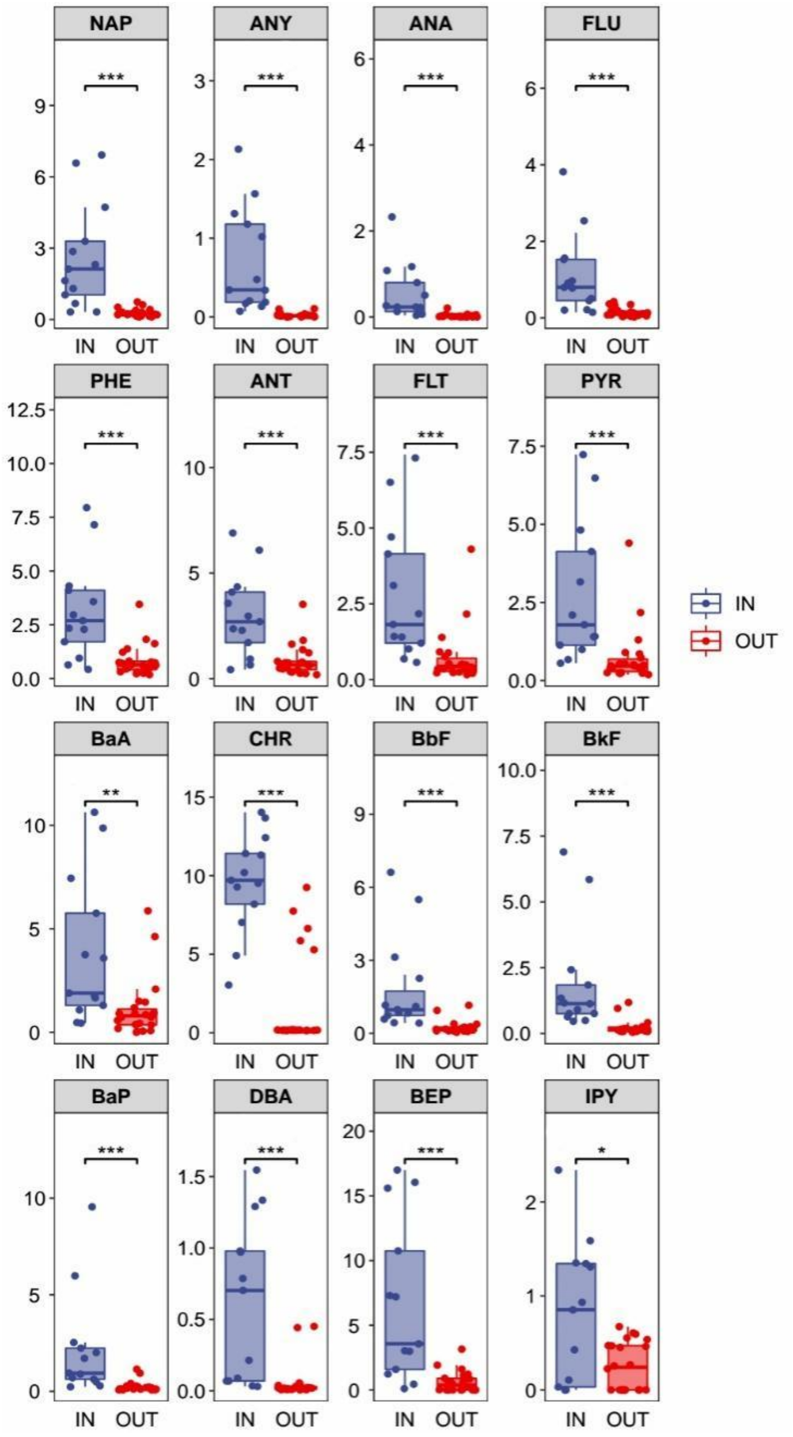

Fig. S3 Box plot showing the distribution of the measured concentrations of 16 Polycyclic aromatic hydrocarbons(mg/kg) in the soil samples inside (IN, blue) and outside (OUT, red) the coking plant (a, c). *** indicate significant differences between two sample areas based on the Wilcoxon signed rank test results at confidence levels of $p < 0.001$.

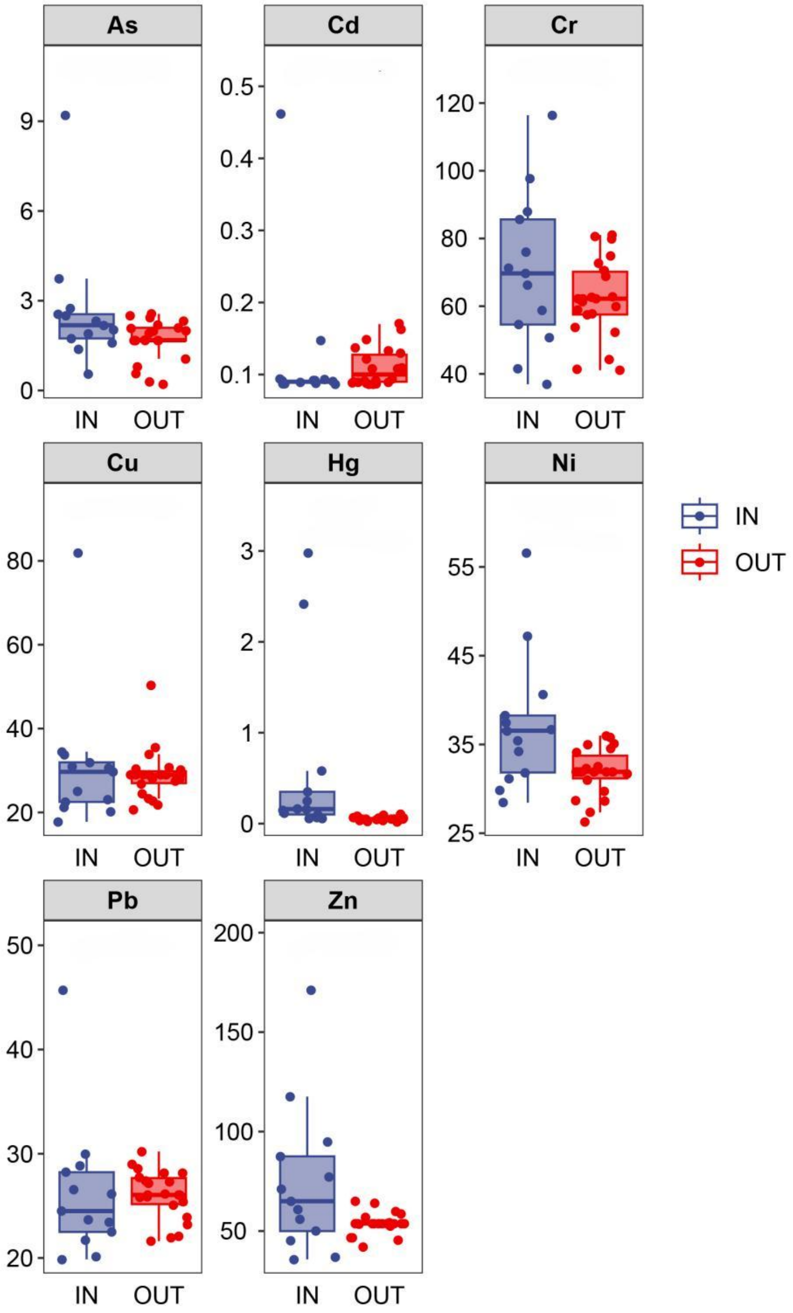

Fig. S4 Box plot showing the distribution of the measured concentrations of the 8 potentially toxic elements (mg/kg) .The concentration of PTEs inside and outside of the plant were not significantly different based on the Wilcoxon signed rank test.

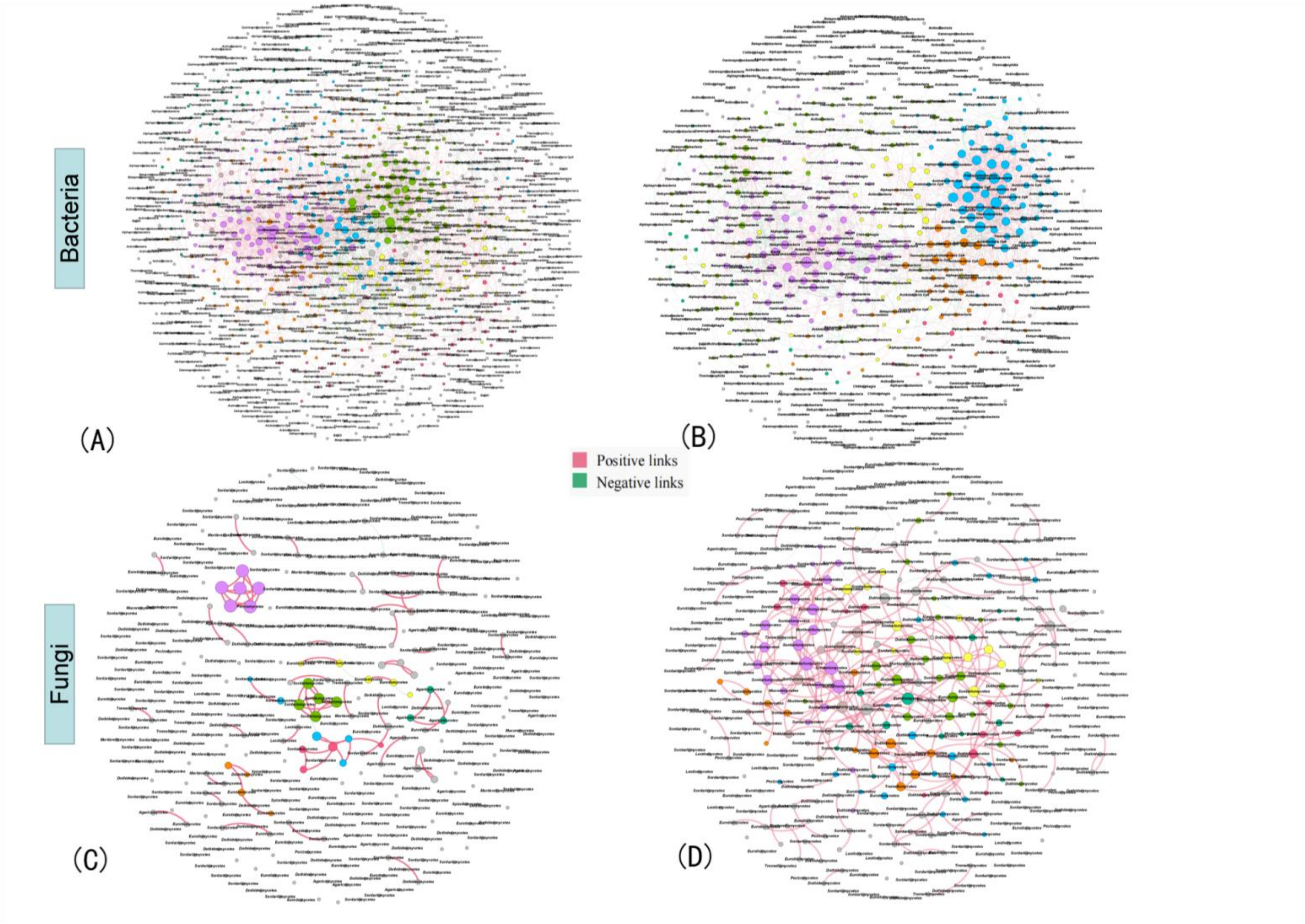

Fig. S5 The networks of soil bacterial (A, B) and fungal (C, D) communities inside(A,C 13 soil samples) and outside(B,D 13 soil samples) the coking plant. The green and red links represent positive and negative correlations, respectively.

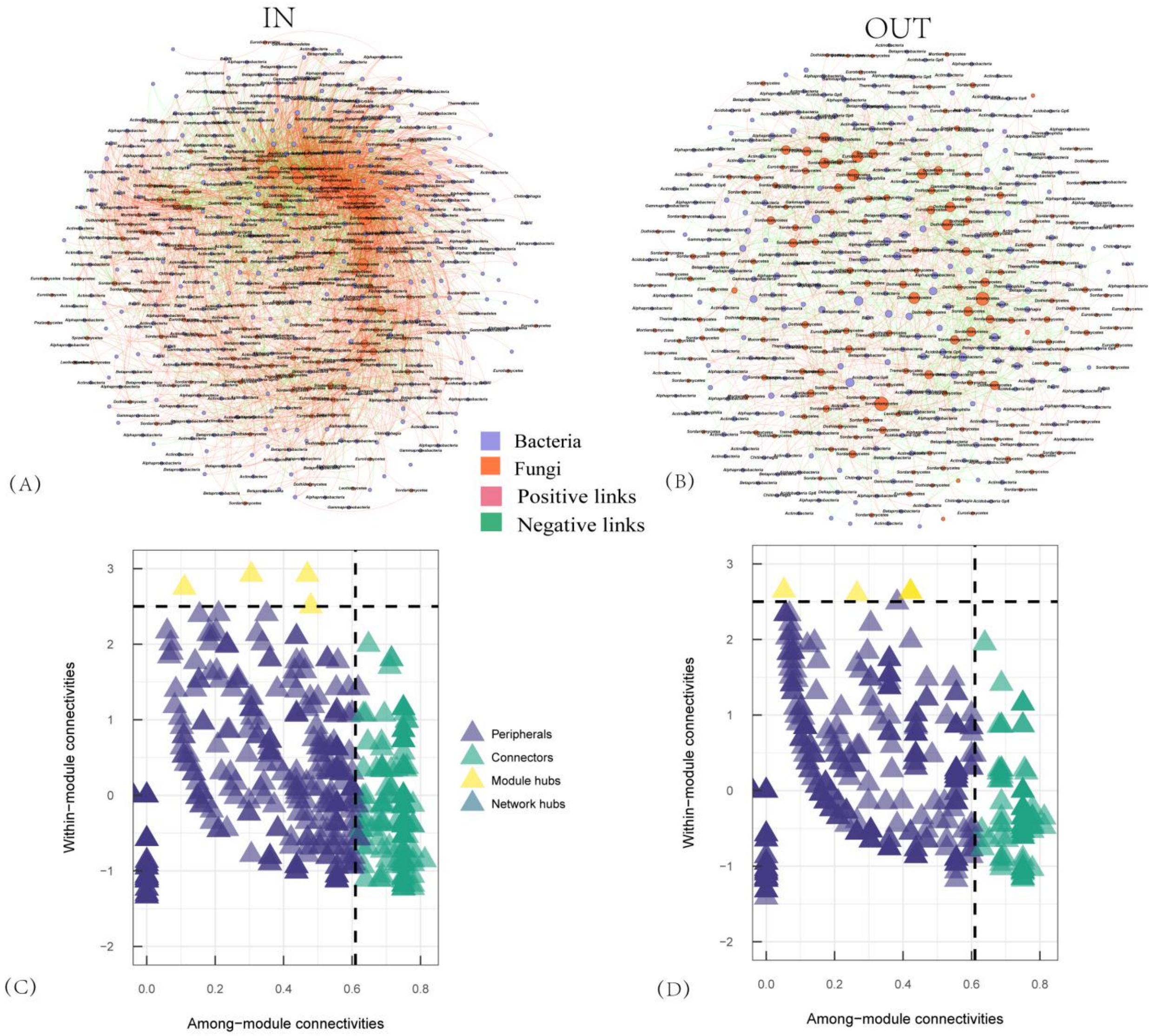

Fig. S6 Interdomain ecological networks of the inside bacterial-fungal associations of inside(A,13 soil samples) and outside(B,13 soil samples) and their Zi-Pi plots of the soil samples inside(C) and outside(D).

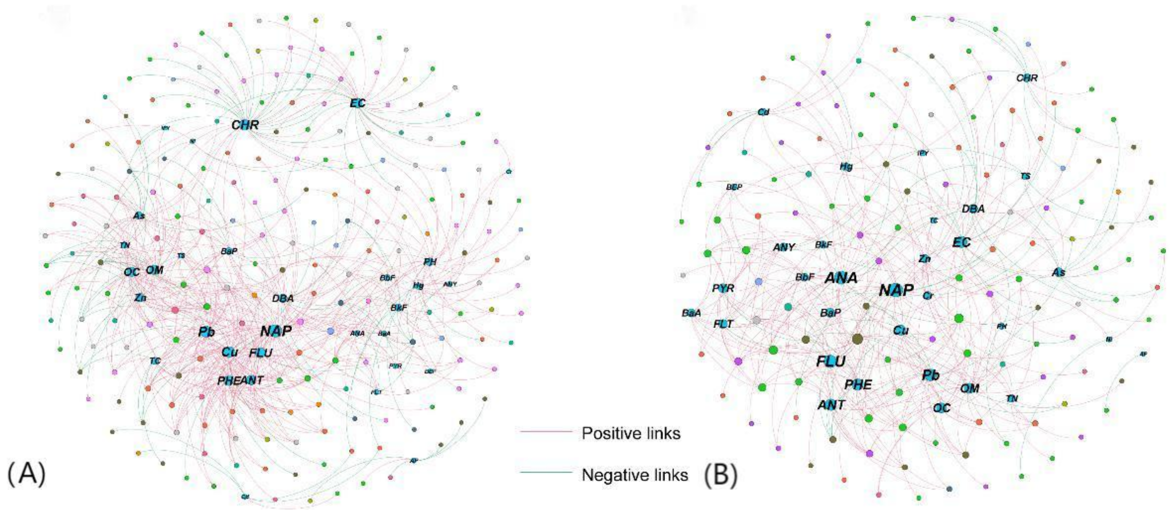

Fig.S7 The bacterial (A) and fungal (B) network inside the coking plant constructed with PAHs-PTEs and soil variables.

1 Tables

Table S1 Soil background values of eight potentially toxic elements (PTEs)elements in Hebei Province, China

	Background value b	
	Hebei province	China
Cd(mg/kg)	0.094	0.03
Hg(mg/kg)	0.036	0.002
As(mg/kg)	13.6	0.01
Pb(mg/kg)	21.5	1.4
Cr(mg/kg)	68.3	0.5
Cu(mg/kg)	21.8	0.4
Zn(mg/kg)	78.4	1.2
Ni(mg/kg)	25	20

Table S2 Soil physicochemical properties inside(IN) and outside(OUT) the coking plant.

		Max		Min		Mean		STD	
		Inside	Outside	Inside	Outside	Inside	Outside	Inside	Outside
pH	–	8.58	8.37	7.54	5.27	8.05	7.14	0.33	0.8
TOC	(g/kg)	49.2	36.8	14.1	7.16	25.27	16.55	12.1	6.86
TN	(g/kg)	3.25	1.72	0.07	0.03	0.89	0.59	0.86	0.42
TC	(g/kg)	189	37.6	15.2	7.87	52.08	16.48	47.2	6.65
TS	(g/kg)	8.9	0..75	0.23	0.11	1.87	0.24	2.37	0.15
OM	(g/kg)	85.3	63.4	24.3	12.35	43.7	28.54	20.9	11.8
AP	(g/kg)	16.2	149	2.54	9.27	5.5	50.87	3.75	35.4
EC(ORP)	(ms/cm)	1.56	0.96	0.17	0.1	0.65	0.22	0.54	0.18
Soil water content	（%）	12.79	27.4	2.75	3，91	8.33	7.82	3.07	4.86

Table S3 Topological properties of the bacterial and fungal molecular ecological networks of soil samples inside (IN,13 soil samples) and outside (OUT,22 soil samples) the coking plant.

Network Indexes	Bacteria_IN	Bacteria_OUT	Fungi_IN	Fungi_OUT
Similarity threshold	0.860	0.860	0.79	0.79
Total nodes	1264	465	371	242
Total links	1733	783	632	213
Positive links (%)	72.5	82.84	31.72	40.38
Negative links (%)	27.5	17.16	68.28	59.62
Modularity	0.602	0.674	0.649	0.877
Average degree (avgK)	3.742	3.368	3.407	1.76
Average clustering coefficient (avgCC)	0.166	0.157	0.114	0.028
Average path distance (GD)	5.973	5.086	5.032	7.087
Maximal degree	25	26	17	8
Density (D)	0.002	0.007	0.009	0.007

Table S4 Topological properties of the bacterial and fungal molecular ecological networks of soil samples inside (IN,13 soil samples) and outside (OUT,13 soil samples) the coking plant.

.Network Indexes	Bacteria_IN	Bacteria_OUT	Fungi_IN	Fungi_OUT
Similarity threshold	0.860	0.860	0.79	0.79
Total nodes	1169	626	434	386
Total links	2897	1882	82	290
Positive links (%)	76.46	75.35	93.3	92.07
Negative links (%)	23.54	24.65	6.1	7.93
Modularity	0.637	0.601	0.932	0.829
Average degree (avgK)	2.478	3.006	0.189	0.751
Average clustering coefficient (avgCC)	0.324	0.384	0.725	0.312
Average path distance (GD)	5.99	4.94	1.25	7.5
Maximal degree	21	15	4	21
Density (D)	0.004	0.01	0.001	0.004
